# Prediction of RNA secondary structure by maximizing pseudo-expected accuracy

**DOI:** 10.1186/1471-2105-11-586

**Published:** 2010-11-30

**Authors:** Michiaki Hamada, Kengo Sato, Kiyoshi Asai

**Affiliations:** 1 Graduate School of Frontier Sciences, The University of Tokyo,5-1-5 Kashiwanoha, Kashiwa, Chiba 277-8562, Japan; 2 Computational Biology Research Center, National Institute of Advanced Industrial Science and Technology (AIST),2-4-7 Aomi, Koto-ku, Tokyo 135-0064, Japan

## Abstract

**Background:**

Recent studies have revealed the importance of considering the entire distribution of possible secondary structures in RNA secondary structure predictions; therefore, a new type of estimator is proposed including the maximum expected accuracy (MEA) estimator. The MEA-based estimators have been designed to maximize the expected accuracy of the base-pairs and have achieved the highest level of accuracy. Those methods, however, do not give the single best prediction of the structure, but employ parameters to control the trade-off between the sensitivity and the positive predictive value (PPV). It is unclear what parameter value we should use, and even the well-trained default parameter value does not, in general, give the best result in popular accuracy measures to each RNA sequence.

**Results:**

Instead of using the expected values of the popular accuracy measures for RNA secondary structure prediction, which is difficult to be calculated, the *pseudo*-expected accuracy, which can easily be computed from base-pairing probabilities, is introduced. It is shown that the pseudo-expected accuracy is a good approximation in terms of sensitivity, PPV, MCC, or F-score. The pseudo-expected accuracy can be approximately maximized for each RNA sequence by stochastic sampling. It is also shown that well-balanced secondary structures between sensitivity and PPV can be predicted with a small computational overhead by combining the pseudo-expected accuracy of MCC or F-score with the γ-centroid estimator.

**Conclusions:**

This study gives not only a method for predicting the secondary structure that balances between sensitivity and PPV, but also a general method for approximately maximizing the (pseudo-)expected accuracy with respect to various evaluation measures including MCC and F-score.

## Background

To predict the secondary structure of an RNA sequence is a classic problem of sequence analysis in bioinformatics. The importance of accurate predictions of secondary structures has increased due to the recent finding of functional non-coding RNAs whose functions are closely related to their secondary structures [1-3]. Secondary structure prediction also plays an important role in research on viral RNAs [4].

There are many tools and algorithms for secondary structure prediction [5-11]. The most popular approach is to predict the minimum free energy (MFE) structure by using the Zuker algorithm [12]. Well-known software (Mfold [13], RNAfold [14] and RNAstructure [15]) employs this approach. From a probabilistic viewpoint, the MFE structure is equivalent to the secondary structure of the maximum likelihood (ML) estimation for the probability distribution of secondary structures given by the McCaskill model [16]. It is, however, known that the MFE/ML structure has drawbacks: there are a huge number of suboptimal structures whose free energies are similar to the minimum free energy and the probability of the MFE structure is extremely small [17]. Moreover, the ML-estimator is not optimized for ac- curacy measures in the target problem [10].

Therefore, another approach that considers the entire distribution of possible secondary structures of a given sequence has been introduced. Ding *et al*. [18] proposed the centroid estimator, which minimizes the expected Hamming loss. On the other hand, Do *et al*. [7] proposed the maximum expected accuracy (MEA) estimator, which gives a prediction based on maximizing the expected value of an accuracy function under a probabilistic distribution of secondary structures. The MEA-based estimators have been applied to many problems in bioinformatics, including sequence analyses for RNA sequences [6,7,10,19-22], alignment of bio- logical sequences [23-25] and other estimation problems [26-28].

For RNA secondary structure predictions, two MEA-based estimators have been proposed: (i) the estimator proposed by [7] and (ii) the γ-centroid estimator proposed by [10]. Both estimators do *not *employ the accuracy measures that are used in actual evaluation of RNA secondary structure, namely, sensitivity (SEN), positive predictive value (PPV), Matthews correlation coefficient (MCC) and F-score, with respect to predicted base-pairs. From a view- point of MEA, it is useful to consider the estimators that maximize expectation of those accuracy measures. Because the computation of those estimators generally demands huge computational time, the previous studies could not use those accuracy measures directly.

Moreover, the previous MEA-based estimators contain a parameter that controls the balance between SEN and PPV of base-pairs in a predicted secondary structure. It is, however, unclear how to select the parameter in order to obtain one reasonable secondary structure (e.g., a well-balanced secondary structure between SEN and PPV), although there are situations that only one predicted secondary structure is required. There is also a possibility that the optimal parameter might depend on the length of sequence and/or the type of RNA family, although the γ-centroid estimator (and the estimator proposed by [7]) employs a default parameter, determined by a benchmark dataset, which is identical for all sequences.

In this study, to address the drawbacks of the current MEA-based methods described above, We introduce the *pseudo*-expected accuracy of a secondary structure with respect to a given accuracy measure, which is a function of the number of true positive base-pairs (TP), true-negative base- pairs (TN), false-positive base-pairs (FP) and false- negative base-pairs (FN). The pseudo-expected accuracy is then defined by using expected TP, TN, FP and FN. As the accuracy measures, we utilize SEN, PPV, MCC and F-score with respect to base-pairs, which are commonly used in the evaluations of RNA secondary structure predictions, because the base- pairs are essential for forming secondary/tertiary structures, which are known to be biologically important.

The pseudo-expected accuracy is easily calculated using the base-pairing probability matrix, and can be computed much more efficiently than the expected accuracy. Although the pseudo-expected accuracy is not equal to the expected accuracy of a predicted secondary structure, we found that the pseudo-expected accuracy gives a good approximation of the expected accuracy in our situation. Accordingly, we also introduce the approximated estimators that maximize the expected accuracy of a given accuracy measure. Moreover, by combining the pseudo-expected MCC/F-score with the γ- centroid estimator, it is possible to predict the balanced secondary structure between SEN and PPV (which seems to be a reasonable secondary structure in many situations when only one predicted secondary structure is required), although there is a small computational overhead.

The techniques described in this paper will be extended to design the maximum expected accuracy estimator for various evaluation measures (cf. [29]).

## Methods

In the following, we represent a secondary structure of an RNA sequence *x *as a triangular binary matrix:*θ *= {*θ*_*ij*_}_*i < j *_where *θ*_*ij *_= 1 means that *i*-th base *x*_*i *_and *j*-th base *x*_*j *_form a base-pair, and *θ*_*ij *_= 0 means that *i*-th base *x*_*i *_and *j*-th base *x*_*j *_do not form a base-pair. In this study, pseudo-knotted base-pairs are not allowed in a secondary structure. For an RNA sequence $x,S(x)(\subset \{\theta_{ij}\in \{0,1\}|1\leq i<j\leq |x|\})$ denotes the space of all the possible secondary structures of *x*, where |*x| *is the length of *x*. A probability distribution on *S*(*x*) (denoted by *p*(·*|x*)) is given by the McCaskill [16], CONTRAfold [7] or Simfold [11] models. The base-pairing probability matrix of *x*, {*p*_*i,j*_}_*i *<*j*_, has entries $p_{ij}=\sum_{\theta \in S(x)}I\left(\theta_{ij}=1\right)p\left(\theta |x\right),$ called base-pairing probabilities, where *I*(·) is the indicator function that returns 1 if the condition is true and 0 otherwise. The base-pairing probability matrix of a given RNA sequence *x *can be computed using the McCaskill (Inside-Outside) algorithm, whose complexities are *O*(*|x|*^3^) and *O*(*|x|*^2^) for time and space, respectively (e.g., see [16,30]).

### Expected accuracy and pseudo-expected accuracy of RNA secondary structure

#### Accuracy measures for RNA secondary structure prediction

For two secondary structures *θ *= {*θ*_*ij*_}_*i < j *_∈ *S*(*x*) and σ = {σ_*ij*_}_*i < j *_∈ *S*(*x*) of an RNA sequence *x*, we define

(1) $$\text{TP}=\text{TP}(\theta ,\sigma )=\sum_{i<j}I(\sigma_{ij}=1)I(\theta_{ij}=1),$$

(2) $$\text{TN}=\text{TN}(\theta ,\sigma )=\sum_{i<j}I(\sigma_{ij}=0)I(\theta_{ij}=0),$$

(3) $$\text{FP}=\text{FP}(\theta ,\sigma )=\sum_{i<j}I(\sigma_{ij}=1)I(\theta_{ij}=0),$$

(4) $$\text{FN}=\text{FN}(\theta ,\sigma )=\sum_{i<j}I(\sigma_{ij}=0)I(\theta_{ij}=1),$$

(5) $$\text{SEN}=\text{SEN}(\theta ,\sigma )=\frac{\text{TP}}{\text{TP}+\text{FN}},$$

(6) $$\text{PPV}=\text{PPV}(\theta ,\sigma )=\frac{\text{TP}}{\text{TP}+\text{FP}},$$

(7) $$\text{MCC }=\text{ MCC}(\theta ,\sigma )=\frac{\text{TP}\cdot \text{TN}-\text{FP}\cdot \text{FN}}{\sqrt{\left(\text{TP}+\text{FP})(\text{TP}+\text{FN})(\text{TN}+\text{FP})(\text{TN}+\text{FN}\right)}},$$

(8) $$\text{F-score}=\text{F-score}(\theta ,\sigma )=\frac{2\cdot \text{TP}}{2\cdot \text{TP}+\text{FP}+\text{FN}}.$$

When *θ *is a *reference *(correct) secondary structure and is a *predicted *secondary structure of *x*, Eqs. (1), (2), (3), (4), (5), (6), (7), and (8) are the number of true positive base-pairs, the number of true negative base-pairs, the number of false positive base-pairs, the number of false negative base-pairs, the SEN, the PPV, the MCC and the F-score, respectively. Because the base-pairs in a secondary structure are biologically important, accuracy measures based on base-pairs are useful and SEN, PPV, MCC and F-score are widely-used accuracy measures for secondary structure predictions. Note that MCC and F-score are balanced measures between SEN and PPV. (F-score is equal to a harmonic mean of SEN and PPV.) In the following, *Acc *means one of the SEN, PPV, MCC and F-score.

#### Expected accuracy of secondary structure

Given a probability distribution *p*(*θ|x*) on *S*(*x*), we calculate the expected values of Eq. (1) to Eq. (4).

(9) $$\hat{\text{T}\text{P}}(\sigma )=E_{\theta |x}[\text{TP}(\theta ,\sigma )]=\sum_{i<j}p_{ij}I(\sigma_{ij}=1),$$

(10) $$\begin{array}{l} \hat{\text{TN}}(\sigma )=E_{\theta |x}[\text{TN}(\theta ,\sigma )]=\\ \frac{\left|x|(|x|-1\right)}{2}-\sum_{i<j}I(\sigma_{ij}=1)\\ -\sum_{i<j}p_{ij}+\sum_{i<j}p_{ij}I(\sigma_{ij}=1), \end{array}$$

(11) $$\hat{\text{FP}}(\sigma )=E_{\theta |x}[\text{FP}(\theta ,\sigma )]=\sum_{i<j}\left(1-p_{ij}\right)I(\sigma_{ij}=1),$$

(12) $$\hat{\text{FN}}(\sigma )=E_{\theta |x}[\text{FN}(\theta ,\sigma )]=\sum_{i<j}p_{ij}(1-I(\sigma_{ij}=1)),$$

Where {*p*_*ij*_} indicates the base-pairing probability matrix. Moreover, we calculate the expected accuracy of an accuracy measure *Acc *(*Acc *is equal to SEN, PPV, MCC or F-score) of σ as follows:

(13) $$\begin{array}{c} \hat{Acc}(\sigma )=E_{\theta |x}[Acc(\theta ,\sigma )]\\ =\sum_{\theta \in S(x)}Acc(\theta ,\sigma )p(\theta |x). \end{array}$$

In order to compute the expected *Acc *for a given secondary structure σ (i.e., $\hat{Acc}(\sigma )$), it is necessary to sum over all the secondary structures of the RNA sequence *x *because no efficient algorithm (such as a dynamic programming algorithm) has been reported. The number of candidate secondary structures increases exponentially with the length of the RNA sequence (more precisely, there are roughly 1.8*^L ^*possible structures for a sequence of length *L*), so to compute the expected *Acc *is an intractable problem. Therefore, we approximate it using *stochastic sampling*: For *N *secondary structures ${\left\{\theta^{\left(n\right)}\right\}}_{n=1}^{N}$ given by stochastic sampling [30,31] of secondary structures, we define

(14) $${\hat{Acc}}^{N}(\sigma )=\frac{1}{N}\sum_{1\leq n\leq N}Acc(\theta^{\left(n\right)},\sigma )$$

for $\sigma \in S(x)\text{. }{\hat{Acc}}^{N}(\sigma )$ converges to $\hat{Acc}(\sigma )$ when *N *is sufficiently large by the properties of stochastic sampling. It should be noted that the sample size *N *grows exponentially with the sequence length to ${\hat{Acc}}^{N}(\sigma )$ be a reliable approximation to the expected *Acc *of σ.

#### Pseudo-expected accuracy of secondary structure

In our situation, *Acc *is generally written as a function of TP, TN, FP and FN:

*Acc *= *f*(TP, TN, FP, FN)

Then, for a secondary structure σ, the *pseudo*-expected *Acc *of is defined by

(15) $${\hat{Acc}}^{0}(\sigma )=f(\hat{\text{TP}},\hat{\text{TN}},\hat{\text{FP}},\hat{\text{FN}}).$$

For example, the pseudo-expected MCC is given by

(16) $${\hat{MCC}}^{0}(\sigma )=\frac{\hat{\text{TP}}.\hat{\text{TN}}-\hat{\text{FP}}.\hat{\text{FN}}}{\sqrt{\left(\hat{\text{TP}}+\hat{\text{FP}}\right)\left(\hat{\text{TP}}+\hat{\text{FN}}\right)\left(\hat{\text{TN}}+\hat{\text{FP}}\right)\left(\hat{\text{TN}}+\hat{\text{FN}}\right)}}.$$

If we have the base-pairing probability matrix of *x*, the pseudo-expected *Acc *of σ can be easily computed by using Eqs. (9), (10), (11) and (12) without employing sampling/enumerating algorithms. Although the pseudo-expected *Acc *is *not *equal to the expected *Acc*, we shall see later that the pseudo-expected *Acc *is a good approximation of the expected *Acc *when *Acc *is equal to SEN, PPV, MCC or F-score.

### Prediction of secondary structure by maximizing pseudo-expected accuracy

The γ-centroid estimator [10] for RNA secondary structure prediction is defined by

(17) $$\hat{\sigma }=\underset{\sigma \in S(x)}{\arg\;\text{max}}\left[\gamma \cdot \hat{\text{TP}}(\sigma )+\hat{\text{TN}}(\sigma )\right]$$

where γ *>*0 controls SEN and PPV of a predicted secondary structure. This estimator is suitable when we would like to predict more TP and TN and fewer FP and FN because Eq. (17) is equivalent to

(18) $$\hat{\sigma }=\underset{\sigma \in S(x)}{\arg\text{ max}}\left[\alpha_{1}\hat{\text{TP}}(\sigma )+\alpha_{2}\hat{\text{TN}}(\sigma )-\alpha_{3}\hat{\text{FP}}(\sigma )-\alpha_{4}\hat{\text{FN}}(\sigma )\right]$$

with γ = (α_1 _+ α_4_)/(α_2 _+ α_3_) and α_*k *_≥ 0. Hamada *et al*. [10] show that the secondary structure of the γ-centroid estimator can be calculated by Nussinovstyle dynamic programming.

Eq. (18) indicates that the γ-centroid estimator is not optimized for the actual evaluation measures (cf. SEN, PPV, MCC and F-score). It is useful to introduce the estimator that maximizes expected SEN, PPV, MCC or F-score directly:

(19) $$\hat{\sigma }=\underset{\sigma \in S(x)}{\arg\;\max}\hat{Acc}(\sigma ).$$

It is, however, difficult to compute the expected *Acc *efficiently for given σ and *p*(θ|*x*). Because *Acc *contains products or divisions of TP, TN, FP and FN, no efficient method to compute the estimator Eq. (19) has been found, in contrast to the γ-centroid estimator of Eq. (17). Instead, we consider estimators that maximize *pseudo*-expected *Acc *as follows.

(20) $$\hat{\sigma }=\underset{\sigma \in S(x)}{\arg\;\max}{\hat{Acc}}^{0}(\sigma ).$$

#### Prediction of secondary structure by maximizing pseudo-expected SEN/PPV

The pseudo-expected SEN and PPV of a secondary structure σ can be computed by

(21) $${\hat{\text{SEN}}}^{0}(\sigma )=\frac{\sum_{i<j}p_{ij}I(\sigma_{ij}=1)}{\sum_{i<j}p_{ij}}\text{,}$$

(22) $${\hat{\text{PPV}}}^{0}(\sigma )=\frac{\sum_{i<j}p_{ij}I(\sigma_{ij}=1)}{\sum_{i<j}I(\sigma_{ij}=1)}\text{.}$$

Therefore, the secondary structure given by maximizing pseudo-expected SEN (Eq. (20) with SEN)) is equivalent to the secondary structure that maximizes the sum of base-paring probabilities of the predicted base-pairs. The secondary structure is, therefore, equivalent to the one given by the γ-centroid estimator with a sufficiently large γ [10]. On the other hand, the secondary structure given by maximizing pseudo-expected PPV (Eq. (20) with PPV)) is equivalent to the secondary structure that consists of (only) one base-pair that has the highest base-pairing probability. (The structure does not seem to be useful.) It should be noted that both structures can be easily computed by using the base-paring probability matrix of the target RNA sequence.

#### Prediction of secondary structure by maximizing pseudo-expected MCC/F-score with stochastic sampling (Method M1)

Because of the computational difficulty of computing "argmax" in Eq. (20) with MCC and F-score (see "Discussion" section for more details), we need to evaluate all the secondary structures in *S*(*x*). The number of secondary structures of a given RNA sequence, however, is so large that it is not practical to enumerate all of them. Therefore, we again employ the stochastic sampling of secondary structures and approximate the estimator of Eq. (20) by

(23) $$\hat{\sigma }=\underset{\sigma \in S}{\arg\max}{\hat{Acc}}^{0}(\sigma )$$

where *S *is a set of secondary structures given by stochastic sampling. Note that the computational cost of this estimator is much smaller than that of predictions based on maximizing the expected MCC/F-score. If the pseudo-expected MCC/F-score gives a good approximation of the expected MCC/F-score and we use a sufficiently large sample size, then the estimator in Eq. (23) should be a reliable approximation to the estimator in Eq. (19) that maximizes the expected MCC/F-score.

### Prediction of secondary structure with **γ**-centroid estimator and pseudo-expected MCC/F-score (Method M2)

In the γ-centroid estimator [10] of Eq. (17) implemented in the software CentroidFold [32], there is a parameter γ that adjusts the balance between SEN and PPV. It is, however, unclear how to select the γ parameter that achieves a reasonable structure although there are several situations that only one predicted secondary structure is required. As described in the previous section, we can predict the secondary structures that maximize (pseudo-)expected SEN or PPV, but the well-balanced secondary structure between SEN and PPV will be more useful in many cases than those structures.

Eq. (18), which is equivalent in form to the γ- centroid estimator, indicates that the γ-centroid estimator can *arbitrarily *control the number/ratio of the true predictions and the false predictions by using the parameter. By combining the pseudo-expected MCC/F-score with the γ-centroid estimator, it is possible to predict the balanced secondary structure between SEN and PPV, as follows. First, we compute the base-pairing probability matrix of the given RNA sequence, and then predict a set of secondary structures *S^g ^*of *x *by using the γ-centroid estimators with 17 γ parameters: γ ∈ {2*^k^*: -5 ≤ *k *≤ 10, *k *∈ ℤ} ∪ {6} that were used in our previous paper to obtain the SEN-PPV curve [7,10]. Here, the secondary structure of the γ-centroid estimator with γ ∈ {2*^k^*: 0 <*k *≤ 10, *k *∈ ℤ} ∪ {6} is computed by using Nussinov-style dynamic programming, but the secondary structure of the γ-centroid estimator with γ ∈ {2*^k^*: -5 ≤ *k *≤ 0, *k *∈ ℤ} can be predicted *without *dynamic programming by selecting all the base-pairs whose probability is larger than 1/(γ+ 1) [10]. Second, we select the secondary structure in *S^g ^*that has the best pseudo-expected MCC/F-score:

(24) $$\hat{\sigma }=\;\underset{\sigma \in S^{g}}{\arg\max}{\hat{Acc}}^{0}(\sigma )\text{.}$$

where *Acc *is equal to MCC or F-score. The pseudo-expected MCC/F-score of each secondary structure *σ *∈ *S^g ^*is easily computed because the base-pairing probability matrix has already been computed.

In this case, using the γ-centroid estimator is more suitable than using the MEA-based estimator proposed by [7], which also has a parameter that controls the balance between SEN and PPV, because the latter has a *bias *to MCC and F-score (see [10] for details).

## Results

We conducted all experiments using a Linux OS ma-chine, which has a 2 GHz AMD Opteron Model 246 processor and 4 GB of memory.

### Experimental settings

For the dataset, we utilized the S-151Rfam dataset [7] that contains 151 RNA sequences with reference structures, each of which was taken from a different family in the Rfam database [1] This dataset has been widely used in previous studies of RNA secondary structure prediction, for example, [7,10,11]. For the probability distribution *p*(*θ|x*) of the secondary structures of RNA sequence *x*, we used the CONTRAfold model (version 2.02) [7] and the McCaskill model [16] (in the Vienna RNA pack-age version 1.8.3 [14]). For evaluation measures, we employed SEN, PPV, MCC and F-score with respect to the base-pairs, which are defined by Eqs. (5), (6), (7) and (8), respectively, where σ is a predicted structure and *θ *is a reference structure.

### Comparison between pseudo-expected accuracy and expected accuracy

In this experiment, we compared the pseudo-expected *Acc *(Eq. (15)) with the expected *Acc *(Eq. (13)) where *Acc *is SEN, PPV, MCC or F-score. First, we obtained a set of secondary structures from the S-151Rfam dataset in the following way. For each RNA sequence in the S-151Rfam dataset, we predicted the secondary structures using the γ-centroid estimator [10] (implemented in CentroidFold) with 17 γ parameters, γ ∈ {2*^k^*: *-*5 ≤ *k *≤ 10} ∪ {6} and two models (the McCaskill [16] and CONTRAfold [7] models). Then, duplicate secondary structures were removed from the set. The set of the secondary structures contains various secondary structures, because the γ-centroid estimator with small γ predicts a small number of base-pairs and the one with large γ predicts a large number of base-pairs [10]. As described in the previous section, it is not feasible to compute the expected *Acc *(Eq. (13)) of a given secondary structure, because the number of possible secondary structures is immense. Therefore, we plotted ${\hat{Acc}}^{0}\left(\sigma \right)$ (i.e., pseudo-expected *Acc *of; Eq. (15)) and ${\hat{Acc}}^{1M}\left(\sigma \right)$ (i.e., expected *Acc *of *σ *approximated by 1 M (1,000,000) samples; Eq. (14)) for each secondary structure *σ *in the set of secondary structures.

**Figure 1:**
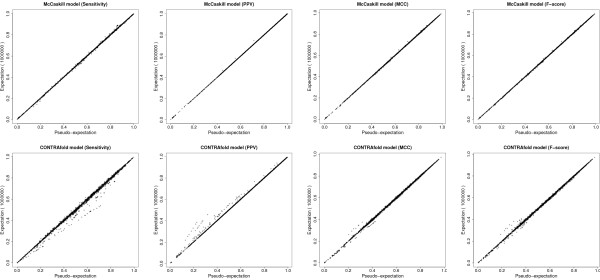
**Comparison between pseudo-expected accuracy and expected accuracy**. Comparison between the *pseudo*-expected SEN, PPV, MCC and F-score (the horizontal axes) and the expected SEN, PPV, MCC and F-score that are computed by stochastic sampling with a sample size of *n *= 1 M (the vertical axes). We used the McCaskill model (top row) and the CONTRAfold model (bottom row). The 1st, 2nd, 3rd and 4th columns indicate SEN, PPV, F-score and MCC, respectively. See Additional file 1, Figure S1 and Figure S2 for other sample sizes.

The result is shown in Figure 1, which indicates the pseudo-expected SEN, PPV, MCC and F-score of the predicted secondary structure is a reliable approximation to the expected SEN, PPV, MCC and F-score, respectively. The averaged squared errors of the pseudo-expected SEN, PPV, MCC and F-score with respect to the CONTRAfold model and the McCaskill model are shown in Additional file 1, Table S1.

### Results of secondary structure prediction by maximizing pseudo-expected accuracy

We conducted the experiments on RNA secondary structure prediction by maximizing the pseudo-expected MCC/F-score of the predicted secondary structure with stochastic sampling (the estimator in Eq. (23)). Note that the results in the previous section suggest that the estimator of Eq. (23) with a sufficiently large sample size is a good approximation to the estimator of Eq. (19) that maximizes the expected MCC/F-score.

**Figure 2:**
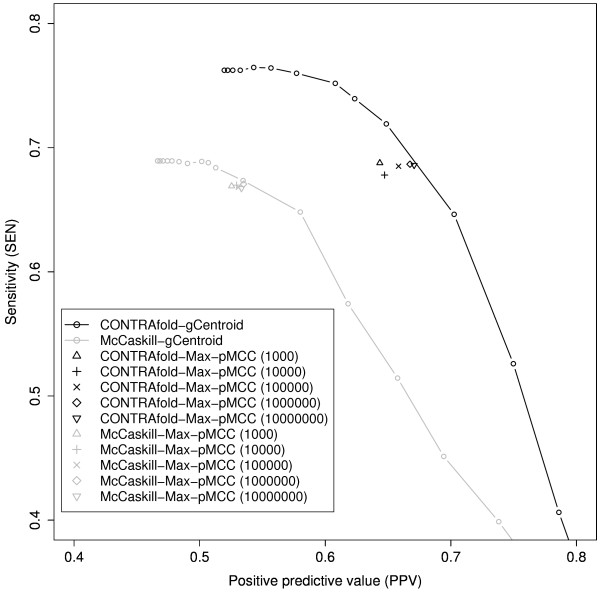
**Performance of RNA secondary structure prediction by maximizing the pseudo-expected MCC with the stochastic sampling (Method M1)**. Performance of RNA secondary structure prediction by "X-Max-pMCC (N)" means the estimator of Eq. (23) with model X and number of samples N with respect to MCC. In the figure, we have also plotted the SEN-PPV curves of the γ-centroid estimator [10] with the CONTRAfold model ("CONTRAfold-gCentroid"; the black line) and with the McCaskill model ("McCaskill-gCentroid"; the gray line). The points and curve in gray and those in black indicate the McCaskill [16] and CONTRAfold [7] models, respectively. See Additional file 1, Figure S3 in the supplementary paper for the results of F-score.

The results are shown in Figure 2 (MCC) and Additional file 1, Figure S1 (F-score). As the sample size increased, the performance of the prediction of the estimator in Eq. (23) converged to the points on the SEN-PPV curves of the γ-centroid estimator [10], and favorable MCC/F-scores were achieved (Table 1). On the other hand, we need to sample a large number of secondary structures (more than 1 million) in order to obtain the secondary structure that has a good MCC/F-score. The computational time of the estimator of Eq. (23) increased linearly with the sample size (Table 2). The result also suggests that it is difficult to improve the performance of the γ-centroid estimator even if we employ the estimator of Eq. (19), that is, maximizing expected MCC/F-score.

**Table 1:** SEN, PPV, MCC and F-score for each prediction algorithm

	McCaskill model				CONTRAfold model			
**γ**	**SEN**	**PPV**	**MCC**	**F-score**	**SEN**	**PPV**	**MCC**	**F-score**

gc-pMCC	0.67	0.54	0.60	0.60	0.68	0.69	0.68	0.68
gc-pF	0.67	0.54	0.60	0.59	0.69	0.68	0.68	0.69
0.03125	0.34	0.78	0.52	0.48	0.09	0.90	0.28	0.16
0.0625	0.40	0.74	0.54	0.52	0.14	0.90	0.36	0.24
0.125	0.45	0.69	0.56	0.55	0.21	0.86	0.42	0.34
0.25	0.51	0.66	0.58	0.58	0.30	0.82	0.50	0.44
0.5	0.57	0.62	0.59	0.60	0.41	0.79	0.56	0.54
1.0	0.65	0.58	0.61	0.61	0.53	0.75	0.63	0.62
2.0	0.67	0.53	0.60	0.60	0.65	0.70	0.67	0.67
4.0	0.68	0.51	0.59	0.59	0.72	0.65	0.68	0.68
6.0	0.69	0.51	0.59	0.58	0.74	0.62	0.68	0.68
8.0	0.69	0.50	0.59	0.58	0.75	0.61	0.68	0.67
16.0	0.69	0.49	0.58	0.57	0.76	0.58	0.66	0.66
32.0	0.69	0.48	0.58	0.57	0.76	0.56	0.65	0.64
64.0	0.69	0.48	0.57	0.56	0.76	0.54	0.64	0.64
128.0	0.69	0.47	0.57	0.56	0.76	0.53	0.64	0.63
256.0	0.69	0.47	0.57	0.56	0.76	0.53	0.63	0.62
512.0	0.69	0.47	0.57	0.56	0.76	0.52	0.63	0.62
1024.0	0.69	0.47	0.57	0.56	0.76	0.52	0.63	0.62

RNAfold	0.65	0.50	0.57	0.57	-			
Simfold	0.64	0.51	0.57	0.57	-			
Sfold	0.65	0.58	0.62	0.62	-			

The rows labeled "gc-pMCC" and "gc-pF" indicate RNA secondary structure prediction with the γ-centroid estimator and the pseudo-expected MCC and F-score, respectively (Method M2). The rows below the dashed line indicate the results of the γ-centroid estimator [10] with various values of the γ parameter (given in the first column). Note that Simfold and Sfold employ a similar probabilistic model (based on energy parameters) for secondary structures to the McCaskill model.

**Table 2:** Computational time in seconds

	Algorithm	McCaskill	CONTRAfold
1	γ-centroid with fixed γ	22	47
2	gCentroid-pMCC	36	59
3	Max-pMCC (1000)	178	303
4	Max-pMCC (10000)	1425	2391
5	Max-pMCC (100000)	13910	23291
6	Max-pMCC (1000000)	138987	232397

Total computational time in seconds for predicting secondary structures of all RNA sequences in the S-151Rfam dataset. The 1st row indicates the γ-centroid estimator [10] with a fixed γ parameter (1 for McCaskill model and 2 for CONTRAfold model). The 2nd row indicates the prediction of RNA secondary structure with the γ-centroid estimator and pseudo-expected MCC (Method M2). "Max-pMCC (N)" from the 3rd to 6th rows indicate the estimator of Eq. (23), that is, RNA secondary structure prediction by maximizing pseudo-expected MCC with stochastic sampling (Method M1) where N is the number of samples.

It should be noted that the performance of the estimator that maximizes the pseudo-expected SEN (PPV) corresponds to the leftmost (rightmost) point in the SEN-PPV curve of the γ-centroid estimators.

### Results of secondary structure prediction with **γ**- centroid estimator and pseudo-expected accuracy

**Figure 3:**
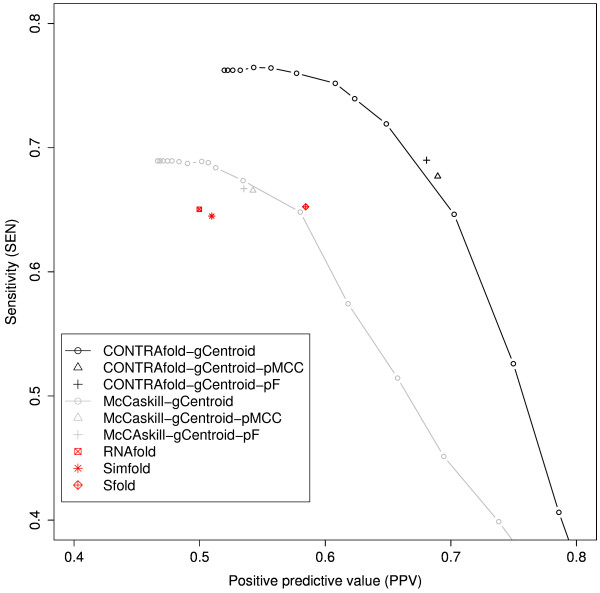
**Performance of RNA secondary structure prediction with the γ-centroid estimator and the pseudo-expected MCC/F-score (Method M2)**. Performance of RNA secondary structure prediction with the γ-centroid estimator and the pseudo-expected MCC (F-score) (the estimator Eq. (24) with MCC (F-score); Method M2); "X-gCentroid-pMCC" ("X-gCentroid-pF") where X is the McCaskill or CONTRAfold model. The curves (X-gCentroid) indicate the performance of the γ-centroid estimator [10] with the McCaskill model and the CONTRAfold model. For comparison, we have also plotted the performance of RNAfold [14], Sfold [5] and Simfold [11] (red points). See Additional file 1, Figure S4 for performances of MEA estimators used in Do *et al*. [7].

Figure 3 shows the performance of RNA secondary structure prediction with the γ-centroid estimator and the pseudo-expected MCC/F-score (Method M2). When the McCaskill model is used, Method M2 is slightly worse than the γ-centroid estimator. However, the performance of Method M2 with the CONTRAfold model is slightly better than the performance of the γ-centroid estimator with the CONTRAfold model. (An example of both pre-dictions is shown in Additional file 1, Figure S5.)

It is also much better than the performance of RNAfold, Sfold and Simfold, all of which return a single prediction. Note that Method M2 with a fixed probabilistic model (e.g., the McCaskill model or the CONTRAfold model) generally achieves performance that differs from that of the γ-centroid estimator with the same model for any γ value. This is because Method M2 automatically selects the secondary structure with the best pseudo-expected MCC/F-score from a set of secondary structures given by the γ-centroid estimator for 17 γ values, while each point in a SEN-PPV curve of the γ- centroid estimator comes from a fixed γ-value.

Table 2 shows that the computational time of Method M2 is much shorter than for Method M1. This is because we do not need to perform any stochastic sampling in Method M2. In Figure 3, we also plotted the performance of Sfold [5], Simfold [11] and RNAfold [14] (the points in red). The results indicate that the secondary structure predicted by Method M2 achieved better accuracy than those methods.

The comparison between the 2nd and 3rd rows in Table 2 indicates that there is only small overhead for the computation of the estimator of Method M2, compared with the γ-centroid estimator with a fixed γ parameter [10]. The reasons can be summarized as follows. The CYK-type algorithm of the Nussinov-style dynamic programming for computing a consistent RNA secondary structure is faster than the Inside-Outside-type algorithm for computing the base-pairing probability matrix in the γ-centroid estimator, even though both algorithms have the same computational complexity. Moreover, we do not need to employ the CYK-type algorithm for the γ- centroid estimator with γ ≤ 1 because we only select the base-pairs whose base-pairing probability is larger than 1/(γ + 1) [10]. Also, the computation of the pseudo-expected MCC/F-score of a given secondary structure is fast enough when the base-pairing probability matrix is computed beforehand.

In summary, by combining the pseudo-expected accuracy with the γ-centroid estimator, we successfully predict the well-balanced secondary structure between SEN and PPV (with small overhead compared to CentroidFold) and the performance (with CONTRAfold model) is better than that of RNAfold, Simfold, Sfold and CentroidFold.

## Discussion and Conclusion

In this study, we introduced the *pseudo*-expected accuracy, (with respect to commonly used accuracy measures in RNA secondary structure prediction: sensitivity, PPV, MCC or F-score) of a given RNA secondary structure under a probability distribution of possible secondary structures. The pseudo-expected accuracy can be computed much more easily than the expected accuracy, because it is computed using the base-pairing probability matrix of the RNA sequence. Although the pseudo-expected accuracy of a given secondary structure is not equal to the expected accuracy of the structure, our computational experiments have indicated that the pseudo-expected accuracy of a given secondary structure is a good approximation of the expected accuracy of the structure when SEN, PPV, MCC and F-score were used as the accuracy mea-sure. This finding is one of the contributions of this study, which has not been reported in previous research.

Based on this finding, we introduced the approximate estimator that maximizes the pseudo-expected accuracy of a prediction by stochastic sampling, which achieved favorable accuracy in our computational experiments. Although the computational cost of this estimator is much smaller than the estimator that maximizes the *expected *accuracy, it is still unacceptably slow. Therefore, we then proposed the combination of the pseudo-expected MCC/F-score and the γ-centroid estimator, which produces one well-balanced secondary structure with small computational overhead. The computational experiments indicate that this approach achieved the best accuracy among state-of-the-art tools. To employ the γ-centroid estimator in Method M2 is suitable because the γ-centroid estimator is able to represent a secondary structures with an arbitrary balance between the expected TP, TN, FP and FN by adjusting the parameter γ (see Eq. (18)). This, however, does not prove that there always exists a γ such that the γ-centroid estimator achieves the *best *pseudo-expected MCC or F-score. Note that the combination of the pseudo-expected MCC/F-score with the MEA-based estimator proposed by [7] is not suitable because the estimator has a *bias *to MCC and F-score, compared to the γ-centroid estimator [10].

Although the trade-ff between SEN and PPV is inherent, and MCC or F-score is not always the best choice of quality measure for predicted secondary structures, the proposed method (Method M2) can be applicable when only a single structure is required. The pseudo-expected MCC/F-score is also employed as a ranking measure of several predicted secondary structures.

### Remarks about terminology: "maximum expected accuracy"

As we described in the Introduction section, the term "maximum (maximizing) expected accuracy" (MEA) has been used in a number of previous studies [6,7,10,26] as well as this study. From a mathematical viewpoint, the MEA (estimator) is a (point) estimator described as follows. Given a predictive space *Y *that contains all the possible candidate solutions of the target problem, a function *Acc*(*θ, y*) for *θ *∈ *Y *and *y *∈ *Y *, and a probability distribution *p*(*θ|D*) on *Y *given data *D*, then the estimator

(25) $$\hat{y}=\underset{y\in Y}{\arg\max}\int Acc(\theta ,y)p(\theta |D)d\theta$$

is introduced. When this estimator is called a "maximum expected accuracy" (MEA) estimator, *Acc*(*θ, y*) is equal to an accuracy measure (or is designed according to an accuracy measure) for a reference *θ *and a prediction *y*. This also implies that *p*(*θ|D*) is considered to be a probability distribution of *references*, which is misleading because *p*(*θ|D*) does not usually represent the distribution. In RNA secondary structure prediction, for example, The McCaskill model provides not a probability distribution of reference secondary structures but rather a full ensemble of possible secondary structures [16].

The estimator of Eq. (25) with a well-designed function *Acc*(*θ, y*) according to accuracy measures for a target problem and a probability distribution *p*(*θ|D*) of solutions empirically achieves better performance than other estimators such as the maximum likelihood estimator and the centroid estimator (i.e., the estimators that minimize the expected hamming difference) in RNA secondary structure predictions [7,10] and in alignments for biological sequences [25].

### Difficulty of computing Eq. (20) with MCC and F-score

Eq. (20) with MCC and F-score can be rewritten as

(26) $$\hat{y}=\underset{\sigma \in S(x)}{\arg\max}\frac{\sum_{i<j}p_{ij}I(\sigma_{ij}=1)}{\sqrt{\sum_{i<j}I(\sigma_{ij}=1)}}\text{ and}$$

(27) $$\hat{y}=\underset{\sigma \in S(x)}{\arg\max}\frac{2\times \sum_{i<j}p_{ij}I(\sigma_{ij}=1)}{\sum_{i<j}I(\sigma_{ij}=1)+\sum_{i<j}p_{ij}},$$

respectively. Note that Eq. (26) is an *approximation *of Eq. (20) with MCC since TN (i.e., the number of true-negative base-pairs) is much larger than the others in RNA secondary structure predictions.

The denominators in both equations prevent division of this optimization problem into sub-problems, which is required to design a dynamic programming algorithm, and hence no efficient algorithms to compute Eqs. (26) and (27) have yet been devised. Note that the "argmax" operation for only the numerator can be efficiently solved by dynamic programming [33]. (This observation does not prove that there exists no efficient (polynomial time) algorithm for computing Eq. (20) with MCC and F-score.)

### The proposed methods are extendable to other situations

We are able to introduce the pseudo-expected ac-curacy for *common *secondary structure prediction of multiple alignments of RNA sequences, because there are several probability distributions for the common secondary structures, for example, the RNAalifold model [34,35] and the Pfold model [36]. Also, the γ-centroid estimator can be extended to common secondary structure prediction [10], and the pseudo-expected MCC/F-score combined with the estimator is useful to predict the common secondary structure that balances between SEN and PPV (See [37]).

Recently, Lu *et al*. [6] proposed the relaxed SEN, PPV and MCC, where slippage of base-pair is al-lowed in computing those measures. It is possible to design the γ-centroid-type estimator that fits with those measures and also to introduce pseudo-expected accuracy of those measures. Moreover, the methods used in this paper can be extended to more general types of estimation problems (cf. [17]) with various accuracy measures that are defined by using TP, TN, FP and FN (cf. [29]).

The method presented in this paper can be applied to local alignments for biological sequences be-cause the γ-centroid estimator was also introduced in the problem [25]. In contrast to *global *alignment problems, the balance between SEN and PPV with respect to aligned bases is important in local alignment problems.

## Authors' contributions

MH and KA conceived the study. MH developed the algorithms, performed the experiments and wrote the manuscript. KS implemented the algorithm in the CentroidFold software. All authors have read and approved the final manuscript.

## Supplementary Material

Additional file 1 **Supplementary Information for the main text**. This file includes supplementary information for the main text. Click here for file
