# Supplementary material for: Prediction of RNA secondary structure by maximizing pseudo-expected accuracy
Source: BMC Bioinformatics. 2010 Nov 30;11:586. doi: 10.1186/1471-2105-11-586 (PMC3003279; doi:10.1186/1471-2105-11-586)
Supplement: Additional file 1 — Supplementary Information for the main text. This file includes supplementary information for the main text. [file 1471-2105-11-586-S1.PDF]

Supplementary Information: Prediction of RNA secondary structure by maximizing pseudo-expected accuracy

Michiaki Hamada*, Kengo Sato and Kiyoshi Asai

Table S1: Averaged squared errors between pseudo-expected accuracy and expected accuracy with stochastic sampling. The column "n" shows the number of stochastic sampling in computing expected accuracy.

	CONTRAfold model				McCaskill model			
n	SEN	PPV	MCC	F-score	SEN	PPV	MCC	F-score
100	5.0e-04	4.8e-04	3.1e-04	3.3e-04	1.2e-04	1.5e-04	1.2e-04	1.1e-04
1000	3.1e-04	1.7e-04	7.5e-05	9.3e-05	2.5e-05	1.8e-05	1.7e-05	1.6e-05
10000	2.7e-04	1.2e-04	4.1e-05	5.8e-05	1.3e-05	2.9e-06	4.5e-06	4.7e-06
100000	2.7e-04	1.2e-04	3.7e-05	5.4e-05	1.1e-05	1.1e-06	3.1e-06	3.3e-06
1000000	2.7e-04	1.2e-04	3.7e-05	5.3e-05	1.1e-05	9.0e-07	2.9e-06	3.1e-06

References

[1] M. Andronescu, A. Condon, H. Hoos, D. Mathews, and K. Murphy. Efficient parameter estimation for RNA secondary structure prediction. *Bioinformatics*, 23:19–28, Jul 2007.

[2] Y. Ding, C. Y. Chan, and C. E. Lawrence. Sfold web server for statistical folding and rational design of nucleic acids. *Nucleic Acids Res*, 32(Web Server issue):135–141, Jul 2004.

[3] C. Do, D. Woods, and S. Batzoglou. CONTRAfold: RNA secondary structure prediction without physics-based models. *Bioinformatics*, 22:e90–98, Jul 2006.

[4] M. Hamada, H. Kiryu, K. Sato, T. Mituyama, and K. Asai. Prediction of RNA secondary structure using generalized centroid estimators. *Bioinformatics*, 25:465–473, Feb 2009.

[5] I. Hofacker, W. Fontana, P. Stadler, S. Bonhoeffer, M. Tacker, and P. Schuster. Fast folding and comparison of RNA secondary structures. *Monatsh. Chem.*, 125:167–188, 1994.

[6] J. S. McCaskill. The equilibrium partition function and base pair binding probabilities for RNA secondary structure. *Biopolymers*, 29(6-7):1105–1119, May 1990.

*Graduate School of Frontier Sciences, The University of Tokyo, 5-1-5 Kashiwanoha, Kashiwa, Chiba 277-8562, Japan
E-mail: `hamada-michiaki@aist.go.jp` or `michiaki.hamada@gmail.com`

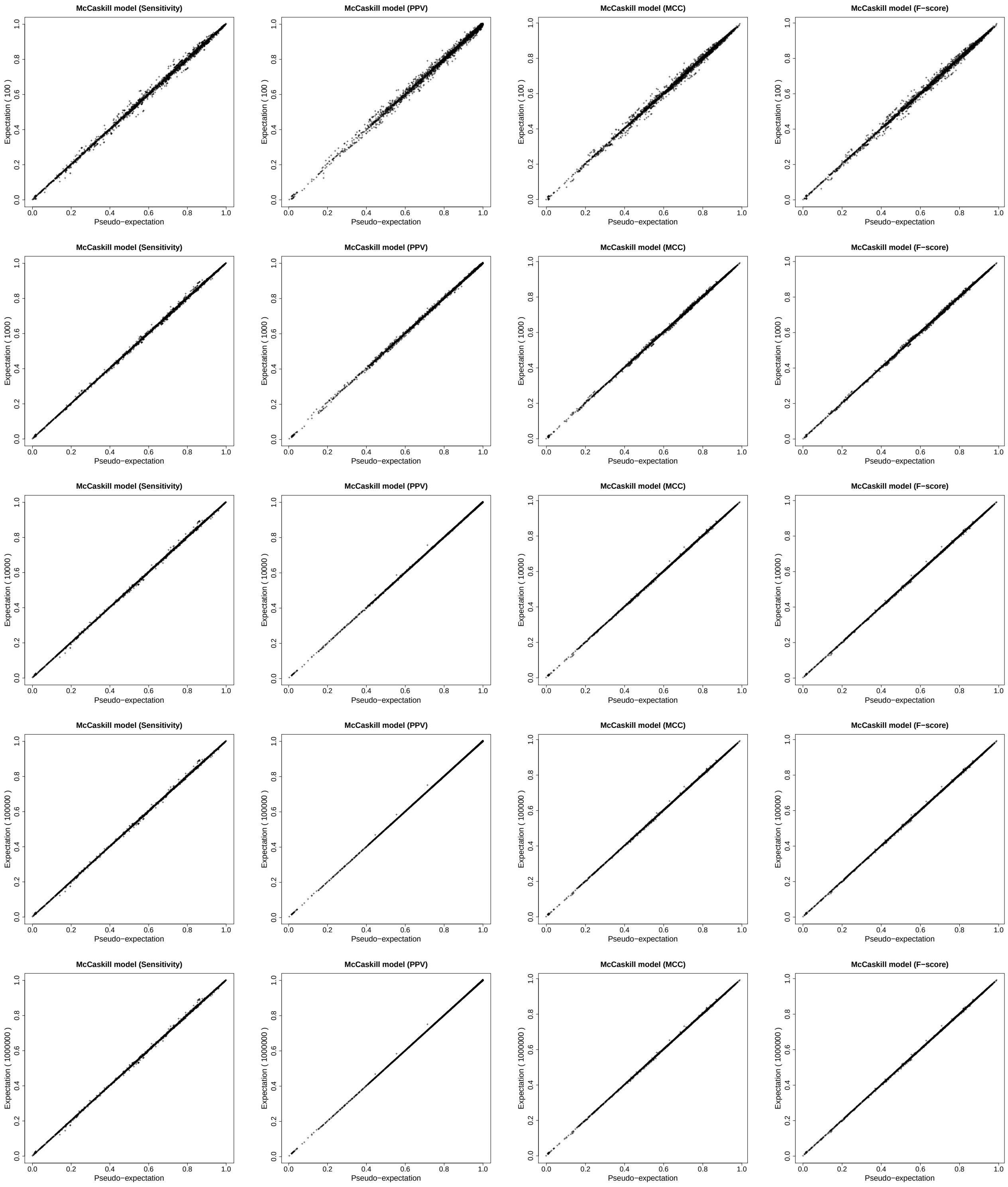

Figure S1: Comparison between the *pseudo*-expected SEN, PPV, MCC and F-score (horizontal axes) and the expected SEN, PPV, MCC and F-score that are computed by stochastic sampling (vertical axes). In this experiment, we used the McCaskill model [6] for a probability distribution of secondary structures. The 1st, 2nd, 3rd, 4th and 5th rows show the sample sizes of 100, 1000, 10000, 100000 and 1000000 in stochastic sampling, respectively. The 1st, 2nd, 3rd and 4th columns indicate sensitivity, PPV, MCC and F-score, respectively.

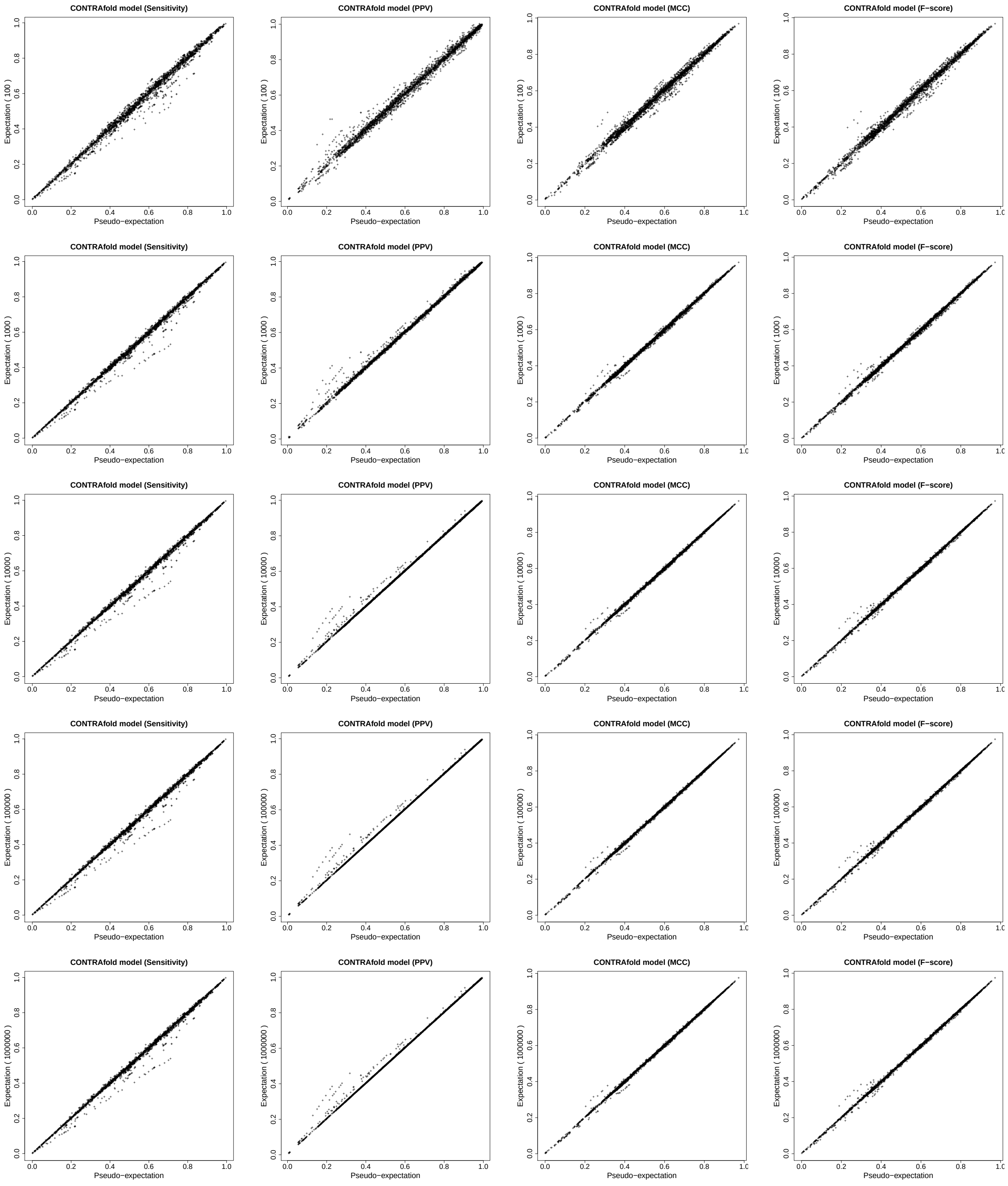

Figure S2: Comparison between the *pseudo*-expected SEN, PPV, MCC and F-score (horizontal axes) and the expected SEN, PPV, MCC and F-score that are computed by stochastic sampling (vertical axes). In this experiment, we used the CONTRAfold model [6] for a probability distribution of secondary structures. The 1st, 2nd, 3rd, 4th and 5th rows show the sample sizes of 100, 1000, 10000, 100000 and 1000000 in stochastic sampling, respectively. The 1st, 2nd, 3rd and 4th columns indicate sensitivity, PPV, MCC and F-score, respectively.

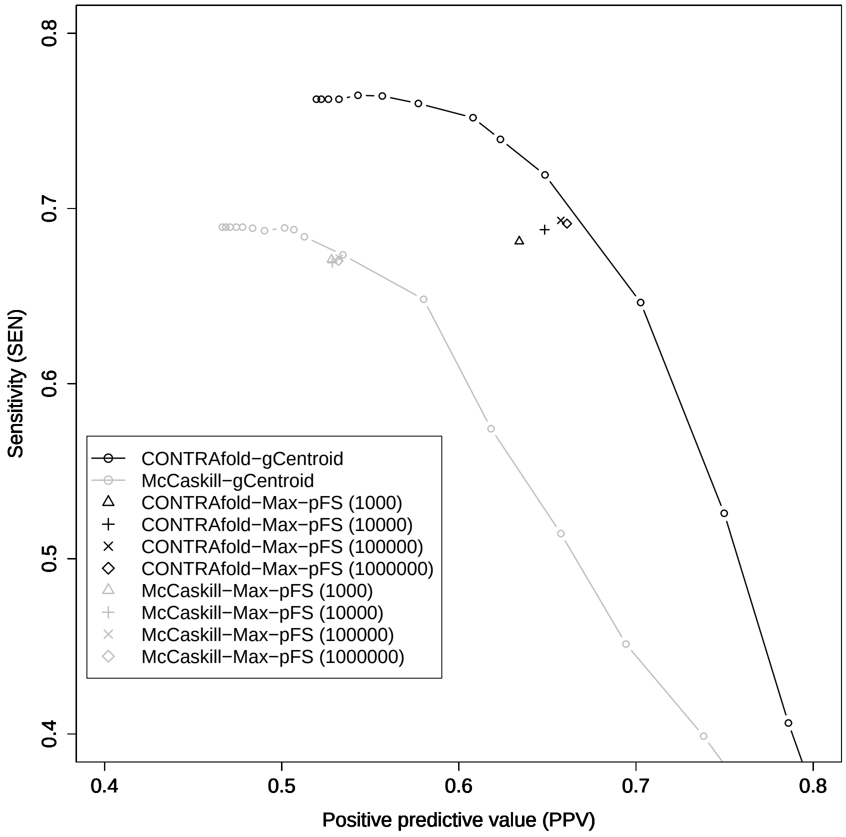

Figure S3: Performances of RNA secondary structure prediction by maximizing the pseudo-expected F-score with the stochastic sampling (Method M1 in the main paper). "X-Max-pFS (N)" means the estimator of Eq. (23) with model X and number of samples N with respect to F-score. In the figure, we have also plotted the SEN-PPV curves of the γ-centroid estimator [4] with the CONTRAfold model ("CONTRAfold-gCentroid"; the black line) and with the McCaskill model ("McCaskill-gCentroid"; the gray line). The points and curve in gray and those in black indicate the McCaskill [6] and CONTRAfold [3] models, respectively.

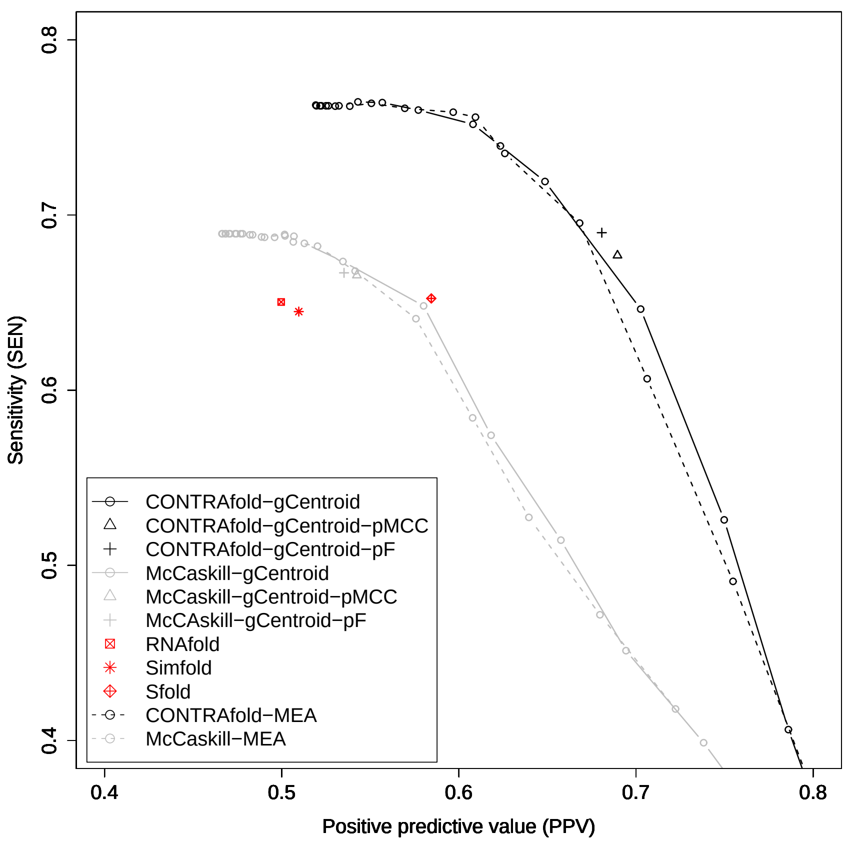

Figure S4: Performance of RNA secondary structure prediction with the γ-centroid estimator and the pseudo-expected MCC (F-score) (the estimator Eq. (24) with MCC (F-score); Method M2); “X-gCentroid-pMCC” (“X-gCentroid-pF”) where X is the McCaskill or CONTRAfold model. The curves (X-gCentroid) indicate the performance of the γ-centroid estimator [4] with the McCaskill model and the CONTRAfold model. For comparison, we have also plotted the performance of RNAfold [5], Sfold [2] and Simfold [1] (red points). The dashed lines indicate the MEA-based estimator proposed by Do *et al.* [3].

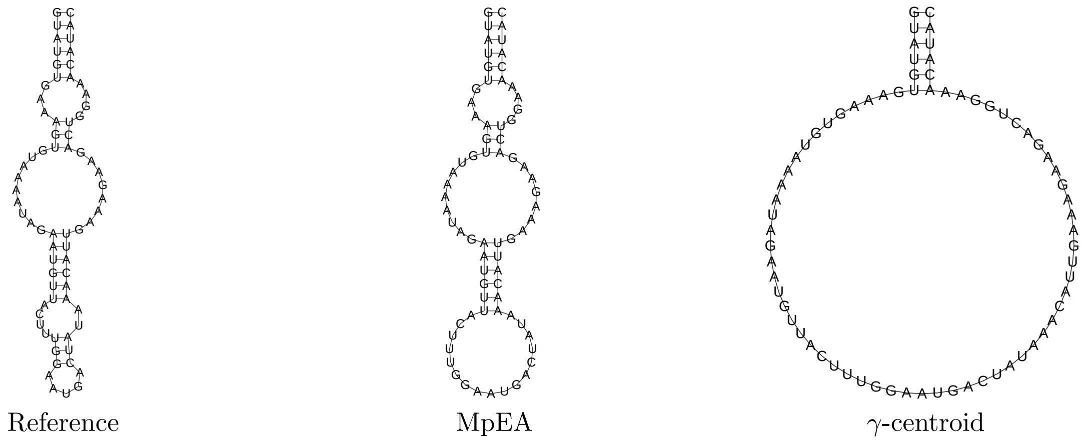

Figure S5: An example of RNA secondary structure prediction (RF00179_B): Reference secondary structure (left), secondary structure of Method M2 with MCC (middle) and secondary structure of γ-centroid estimator with a default parameter: $\gamma = 2$ (right).
